# Evaluation of an antibiotic stewardship program for promoting rational antibiotic use in an ICU in China

**DOI:** 10.1186/s12879-025-11718-4

**Published:** 2025-10-14

**Authors:** Yujin Guo, Lu Kong, Jingjing Liu, Renzhe Li, Mengfei Cui, Xinru Kong, Xue Li, Mengqi Yang

**Affiliations:** 1 Department of Clinical Pharmacy & Pharmacology, Jining No.1 People’s Hospital, Jining, 272011 China; 2 Department of Clinical Laboratory, Jining No.1 People’s Hospital, Jining, 272011 China

**Keywords:** Antimicrobial stewardship programs, Intensive care units, Carbapenems, Multidrug-resistant organisms, Clinical pharmacists

## Abstract

**Background:**

The irrational use of broad-spectrum antibiotics in intensive care units (ICUs) has led to increasing bacterial resistance in recent years. However, research on the effectiveness of antimicrobial stewardship programs (ASPs) in Chinese ICUs is limited. This study aimed to assess the impact of ASP implementation on antibiotic use and bacterial resistance in a Chinese ICU.

**Methods:**

This retrospective, interventional study employed an interrupted time series design and was conducted in an ICU beginning on June 1, 2019. The ASP included the formation of a multidisciplinary team, the development of facility-specific criteria, prescriber education, the implementation of preauthorization processes, and retrospective prescription audit and feedback (RPAF). Patient data were collected from June 1, 2018, to May 31, 2020. An interrupted time series analysis was used to evaluate the impact of the ASP.

**Results:**

A total of 862 patients were admitted during the pre-intervention period, and 946 patients were admitted during the post-intervention period. The interrupted time series analysis demonstrated a reduction in the monthly consumption of carbapenems (β3: −2.25 DDD/100 PD, *p* < 0.001) and linezolid (β3: −0.49 DDD/100 PD, *p* = 0.003) after the intervention. Additionally, a decrease in the incidence of bacteremia caused by multidrug-resistant (MDR) organisms overall (β3:−0.03 events/100 PD, *p* = 0.036) and MDR Gram-positive organisms (β3:−0.01 events/100 PD, *p* = 0.002) was observed post-intervention.

**Conclusion:**

Reduced antimicrobial consumption and decreased incidence of infections associated with MDR organisms were observed following the implementation of an ASP strategy that incorporated clinical pharmacists as core team members.

**Clinical trial number:**

Not applicable.

**Supplementary Information:**

The online version contains supplementary material available at 10.1186/s12879-025-11718-4.

## Introduction

The continuous increase in bacterial resistance in recent years has been closely linked to the irrational and excessive use of broad-spectrum antibiotics [1]. Patients in ICUs, who often have severe underlying conditions, undergo frequent invasive procedures, and experience prolonged hospital stays, are particularly receiving extensive antibiotic therapy [2]. Data on ICU drug consumption indicate that antibiotics frequently rank among the top three classes of drugs administered in ICUs [3]. However, antibiotic use in ICUs is often irrational [4], with the rate of inappropriate carbapenem administration exceeding 60% [5]. Therefore, promoting the rational use of antibiotics, particularly carbapenems, in ICUs remains a significant challenge.

In recent years, the Chinese health authorities have addressed this challenge by introducing a series of antibiotic management policies and guidelines [6, 7], which have been effective in reducing antibiotic consumption in ICUs [8]. However, there is still a lack of effective stewardship programs specifically targeting carbapenem use in China. Furthermore, surveys have shown that carbapenem consumption in Chinese ICUs continues to increase [9]. Alarmingly, recent studies have reported a persistently high detection rate of carbapenem-resistant Enterobacteriaceae (CRE) in these units [10, 11]. Management of carbapenem-resistant organism (CRO) infections, such as those caused by CRE and carbapenem-resistant *Acinetobacter baumannii* (CRAB), poses a serious clinical challenge, associated with limited therapeutic options and high mortality rates [12–14].

To address the threat of drug-resistant bacteria and promote the rational use of antibiotics, several professional societies and international organizations have issued guidelines recommending the implementation of ASPs [15, 16]. Substantial evidence demonstrates that ASPs are effective in promoting judicious antibiotic use and reducing bacterial resistance [17]. However, relatively few reports have described the implementation of ASPs in Chinese ICUs, and the impact of these programs in this setting remains unclear.

This study implemented an ASP in the ICU of a large teaching hospital in China to evaluate its impact on antibiotic use and bacterial resistance. Additionally, the effects of the ASP on antibiotic consumption costs, hospital mortality, length of hospital stay, length of ICU stay, and bloodstream infections caused by MDR organisms were assessed. The study aims to provide practical guidance for antibiotic management in China, with the ultimate goal of promoting the rational use of antibiotics and slowing the emergence of resistant bacteria.

## Methods

### Study design and setting

This retrospective cohort study employed an interrupted time series design. It was conducted in a 38-bed ICU at a tertiary medical institution in Shandong Province, China. The ICU primarily admitted adult patients transferred from general wards, operating rooms, emergency departments, and other facilities. Infection control measures remained unchanged before and after the implementation of the ASP.

Patient characteristics, antibiotic usage, and other outcomes (see Supplementary Table 1) were obtained from the integrated hospital information system (IHIS). Ethical approval was granted by the Research Ethics Board of Jining First People’s Hospital. The requirement for informed consent was waived because the study used anonymized, aggregated, retrospective data.

### Intervention and outcomes measured

This study followed established recommendations for implementing an ASP [18]. The program was co-led by the director of the medical affairs department and the director of the clinical pharmacy department. A multidisciplinary team was formed, comprising one clinical microbiologists, five infectious disease physicians, and five clinical pharmacists. The team developed facility-specific criteria for managing common infectious diseases, based on local epidemiology, susceptibility patterns, and available drugs. These criteria included recommendations for antibiotics such as imipenem/cilastatin, meropenem, fluoroquinolones, piperacillin-tazobactam, cefoperazone-sulbactam, vancomycin, linezolid, and third- and fourth-generation cephalosporins. They were distributed to prescribers via DingTalk (an intelligent mobile office platform). Criteria for prescribing carbapenems were integrated into the IHIS (Supplementary Table 2), requiring prescribers to select the indication at the point of order entry. Clinical pharmacists also provided regular education to ICU prescribers and nurses to ensure they were familiar with these criteria.

The hospital stocked two types of carbapenems: imipenem/cilastatin and meropenem, and implemented a preauthorization system to regulate their use. When a carbapenem prescription was entered into the IHIS, it was initially blocked, triggering a pop-up dialog box requiring the prescriber to select the appropriate indication. The Clinical Pharmacy Department then reviewed the application. If the patient’s condition met the criteria, approval was granted. The reviewing pharmacist specified the type of carbapenem, dosage, treatment duration, and other relevant details, after which the block was lifted. If the pharmacist determined that the criteria were not met, they contacted the prescriber to recommend an alternative antibiotic in accordance with the approved facility criteria. If the prescriber insisted on administering a carbapenem despite not meeting the criteria, the pharmacist authorized the order but recorded it as controversial. Controversial prescriptions were verified monthly by the multidisciplinary team, which assessed (i) whether prescribers had correctly selected indications and (ii) whether pharmacists’ judgements were appropriate. The team also conducted monthly spot checks on approved prescriptions to ensure compliance with carbapenem prescription authorizations.

To ensure competency, strict admission criteria were applied to pharmacists authorized to review carbapenem prescriptions. Requirements included (i) comprehensive knowledge of antibiotics and infectious disease management, (ii) proficiency in interpreting susceptibility testing results, (iii) completion of specialized training in the management of infectious diseases, and (iv) at least 3 years of practical clinical experience in managing infectious diseases. Only pharmacists who passed rigorous examinations were permitted to authorize carbapenem prescriptions. Additionally, ongoing training was provided to improve pharmacists’ skills through in-hospital sessions and exchange programs with other institutions.

Preauthorization of all carbapenem prescriptions submitted via the IHIS occurred from 8:00 a.m. to 5:00 p.m. daily. In emergencies, prescribers were permitted to administer a 24-hour dose of carbapenems before approval.

A retrospective prescription audit and feedback (RPAF) was also conducted for all antibiotic prescriptions, including those for carbapenems. The multidisciplinary team performed random monthly audits of ICU antibiotic prescriptions, and clinical pharmacists communicated feedback directly to prescribers. Pharmacists additionally monitored monthly antibiotic use and associated costs in the ICU. The ASP was implemented in the ICU on June 1, 2019. The pre-intervention period was June 1, 2018, to May 31, 2019, and the post-intervention period was June 1, 2019, to May 31, 2020.

### Outcomes

The primary outcomes were differences in antibiotic consumption and the incidence of bacteremia caused by MDR organisms. Antibiotic consumption was measured as the defined daily doses (DDD) per 100 patient-days (PD), according to the WHO methodology [19]. Incidence of bacteremia was expressed as events per 100 PD, and MDR organisms were identified using standard laboratory methods [20, 21].

Secondary outcomes included (i) hospital mortality rates, expressed as all-cause deaths per 100 PD; (ii) the mean length of hospital stay, (iii) the mean length of ICU stay; and (iv) antibiotic costs, expressed in yuan per 100 PD.

### Statistical analysis

Baseline categorical variables were analyzed using the chi-square test, and continuous variables were assessed with the Mann-Whitney U test. Interrupted time series (ITS) analysis [22] was employed to evaluate monthly antibiotic consumption, the incidence of bloodstream infections, hospital mortality, the mean length of hospital stay, the mean length of ICU stay, and the costs associated with antibiotic use.

The ITS model was specified as:

$$\mathrm{Yt}=\mathrm\beta0+\mathrm\beta1\mathrm{Tt}+\mathrm\beta2\mathrm{Xt}+\mathrm\beta3\mathrm{XtTt}+{\mathrm\varepsilon}_{\mathrm t}$$

Where:

Yt = outcome variable in month t (antibiotic consumption, costs, infections, mortality, mean length of hospital stay, and mean length of ICU stay),

Tt = continuous variable representing the time trend,

Xt = dummy variable for the intervention,

XtTt = interaction term between intervention and time,

β0 = baseline level at time 0,

β1 = pre-intervention trend,

β2 = immediate change post-intervention,

β3 = trend change post-intervention,

εₜ = random error term.

Analyses were based on monthly data from June 1, 2018, to May 31, 2020. All statistical analyses were conducted using IBM SPSS Statistics (IBM, version 25) or Stata (StataCorp, version 15.0), with statistical significance set at *p* < 0.05.

## Results

### Patient characteristics

During the pre-intervention period, 862 patients were admitted to the ICU, compared with 946 during the post-intervention period. No significant differences were observed in baseline characteristics between the two groups. (Supplementary Table 1).

## Changes in antibiotic consumption and costs

**Fig. 1:**
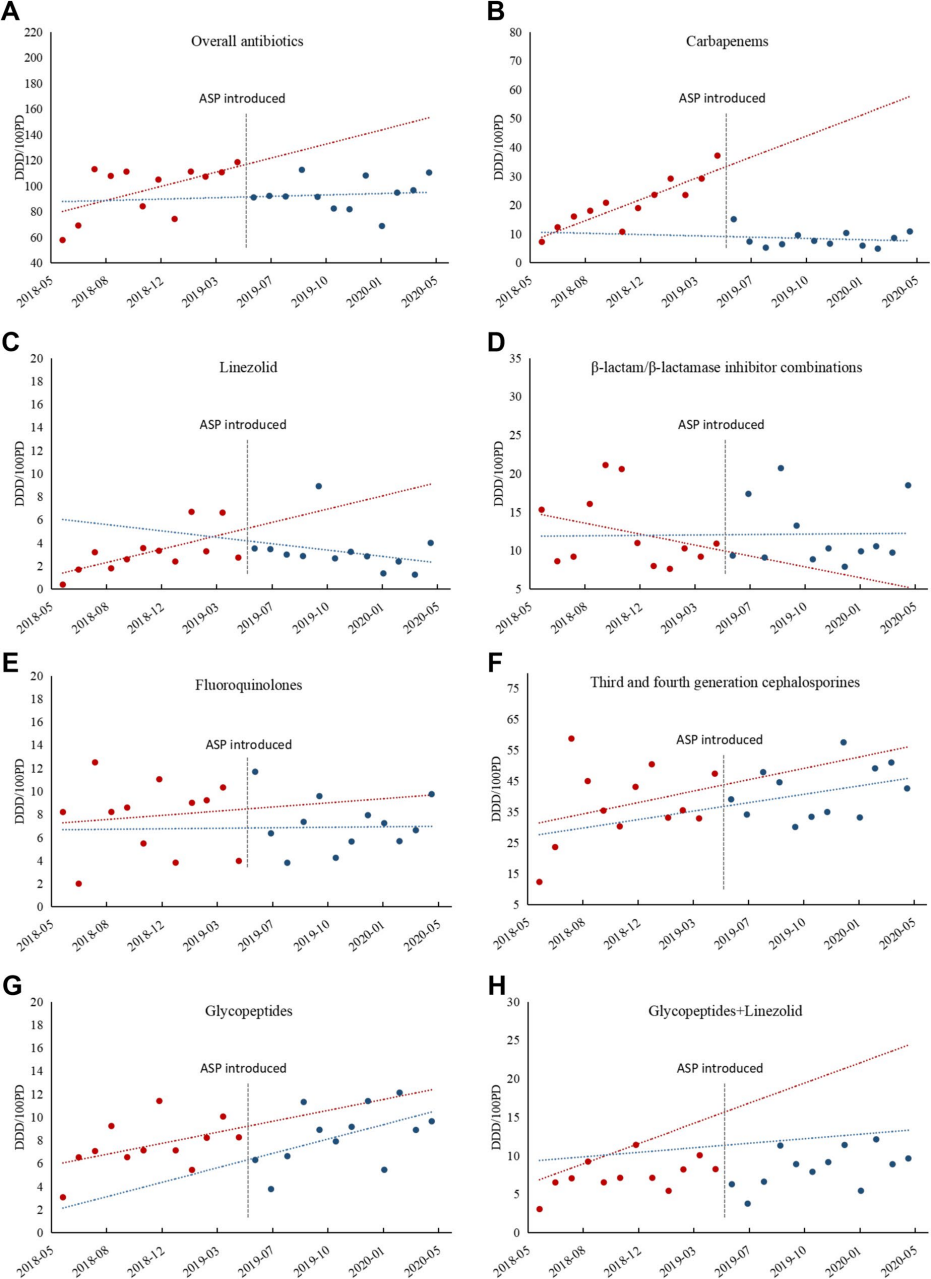
Changes in antibiotic consumption and costs in the ICU pre- and post-intervention. Trends are displayed in red dotted lines (pre-intervention) and blue dotted lines (post-intervention). (**A**) Changes in overall antibiotic consumption; (**B**) Changes in carbapenem consumption; (**C**) Changes in linezolid consumption; (**D**) Changes in β-lactam/β-lactamase inhibitor combination consumption; (**E**) Changes in fluoroquinolone consumption; (**F**) Changes in third- and fourth-generation cephalosporin consumption; (**G**) Changes in glycopeptide consumption; (**H**) Changes in glycopeptide and linezolid consumption

ITS analysis quantified changes in antibiotic use following ASP implementation in the ICU (Table 1; Fig. 1). Before the intervention, significant upward monthly trends were observed for overall antibiotic use (β₁ = 3.18 DDDs/100 PD; 95% CI: 0.59 to 5.77; *p* = 0.019), carbapenems (β₁ = 2.12 DDDs/100 PD; 95% CI: 1.44 to 2.80; *p* < 0.001), and linezolid (β₁ = 0.33 DDDs/100 PD; 95% CI: 0.11 to 0.56; *p* = 0.006). No significant change in pre-intervention trends was observed for β-lactam/β-lactamase inhibitor combinations, fluoroquinolones, third- and fourth-generation cephalosporins, glycopeptides, and the glycopeptide-linezolid category (all *p* > 0.05).

ASP implementation was associated with immediate reductions in overall antibiotic consumption (β₂ = −26.51 DDDs/100 PD; 95% CI: −52.06 to −0.97; *p* = 0.043) and carbapenem use (β₂ = −25.34 DDDs/100 PD; 95% CI: −31.94 to −18.74; *p* < 0.001). A non-significant increase was observed for β-lactam/β-lactamase inhibitor combinations (β₂ = 2.33 DDDs/100 PD; 95% CI: −6.42 to 11.08; *p* = 0.585), while other antibiotic categories showed no significant immediate changes post-intervention.

Significant sustained monthly post-intervention reductions were observed for carbapenems (β₃ = −2.25 DDDs/100 PD; 95% CI: −3.23 to −1.27; *p* < 0.001) and linezolid (β₃ = −0.49 DDDs/100 PD; 95% CI: −0.81 to −0.18; *p* = 0.003). Overall antibiotic use showed a non-significant downward trend (β₃ = −2.86 DDDs/100 PD; 95% CI: −6.50 to 0.78; *p* = 0.117). Notably, no significant long-term trend changes were observed for β-lactam/β-lactamase inhibitor combinations, fluoroquinolones, third- and fourth-generation cephalosporins, glycopeptides, and the glycopeptide-linezolid category.

**Table 1:** Effect of the ASP on antibiotic consumption in the ICU

Antibiotics	Parameter evaluated	Effect of intervention	Standard Error	t	*P*	95% CI
Overall antibiotics	Pre-Intervention Trends (β1)	3.18	1.24	2.56	0.019	(0.59,5.77)
	Immediate Level Changes (β2)	−26.51	12.24	−2.17	0.043	(− 52.06, − 0.97)
	Pro-Intervention Trends (β3)	−2.86	1.74	−1.64	0.117	(− 6.50, 0.78)
Carbapenems	Pre-Intervention Trends (β1)	2.12	0.33	6.49	0.000	(1.44,2.80)
	Immediate Level Changes (β2)	−25.34	3.16	−8.01	0.000	(− 31.94, − 18.74)
	Pro-Intervention Trends (β3)	−2.25	0.47	−4.81	0.000	(− 3.23, − 1.27)
Linezolid	Pre-Intervention Trends (β1)	0.33	0.11	3.10	0.006	(0.11,0.56)
	Immediate Level Changes (β2)	−1.30	1.08	−1.21	0.240	(− 3.55, 0.94)
	Pro-Intervention Trends (β3)	−0.49	0.15	−3.32	0.003	(− 0.81, − 0.18)
β-lactam/β-lactamase inhibitor combinations	Pre-Intervention Trends (β1)	−0.41	0.44	−0.93	0.362	(− 1.33,0.51)
	Immediate Level Changes (β2)	2.33	4.19	0.55	0.585	(− 6.42, 11.08)
	Pro-Intervention Trends (β3)	0.43	0.64	0.67	0.512	(− 0.91, 1.76)
Fluoroquinolones	Pre-Intervention Trends (β1)	0.10	0.15	0.71	0.489	(− 0.20,0.41)
	Immediate Level Changes (β2)	−1.69	1.48	−1.14	0.266	(− 4.78, 1.39)
	Pro-Intervention Trends (β3)	−0.09	0.20	−0.45	0.657	(− 0.52, 0.33)
Third- and fourth-generation cephalosporins	Pre-Intervention Trends (β1)	1.07	0.90	1.19	0.248	(− 0.80,2.94)
	Immediate Level Changes (β2)	−7.13	8.79	−0.81	0.427	(− 25.47, 11.22)
	Pro-Intervention Trends (β3)	−0.28	1.27	−0.22	0.829	(− 2.92, 2.37)
Glycopeptides	Pre-Intervention Trends (β1)	0.28	0.15	1.80	0.087	(− 0.04,0.60)
	Immediate Level Changes (β2)	−2.87	1.52	−1.88	0.074	(− 6.05, 0.31)
	Pro-Intervention Trends (β3)	0.09	0.21	0.40	0.690	(− 0.36, 0.53)
Glycopeptides + Linezolid	Pre-Intervention Trends (β1)	0.76	0.150	1.80	0.087	(−0.040,0.60)
	Immediate Level Changes (β2)	−2.88	1.52	−1.88	0.07	(−6.05,0.31)
	Pro-Intervention Trends (β3)	0.09	0.21	0.40	0.69	(−0.36,0.53)

The ASP was also associated with significant reductions in antibiotic costs (Table 2). Before the intervention, costs demonstrated a significant upward trend (β₁ = 1244.21 Yuan/100 PD; 95% CI: 585.91 to 1902.50; *p* = 0.001). After ASP implementation, although the immediate cost reduction was not statistically significant (level change β₂ = −3393.87 Yuan/100 PD; 95% CI: −9208.61 to 2420.88; *p* = 0.238), a significant downward trend emerged post-intervention with monthly cost reductions averaging β₃ = −2160.69 Yuan/100 PD (95% CI: −2969.13 to −1352.26; *p* < 0.001).

**Table 2:** Effect of the ASP on antibiotic costs in the ICU

	Parameter evaluated	Effect of intervention	Standard Error	t	*P*	95% CI
Antibiotics costs	Pre-Intervention Trends (β1)	1244.21	315.59	3.94	0.001	(585.91,1902.5)
	Immediate Level Changes (β2)	−3393.87	2787.56	−1.22	0.238	(− 9208.61,2420.88)
	Pro-Intervention Trends (β3)	−2160.69	387.56	−5.58	0.000	(− 2969.13,−1352.26)

### Incidence rate of infections caused by MDR organisms

**Fig. 2:**
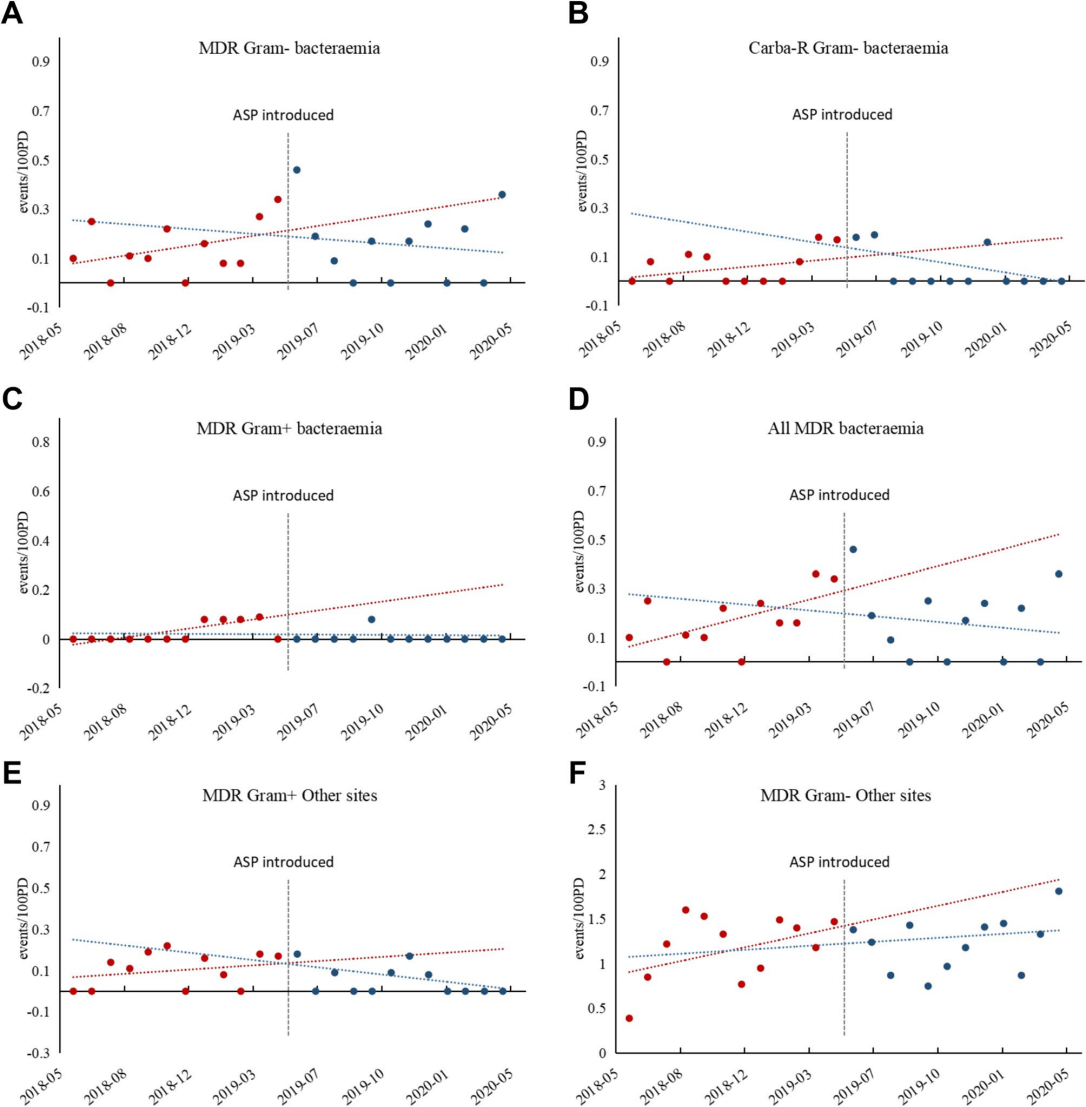
Changes in the incidence of bacteremia and other infections caused by multi-drug resistant organisms in the ICU pre- and post-intervention. Trends are displayed in red dotted lines (pre-intervention) and blue dotted lines (post-intervention). (**A**) Changes in the incidence of bacteremia caused by MDR Gram − organisms; (**B**) Changes in the incidence of bacteremia caused by Carba-R Gram − organisms; (**C**) Changes in the incidence of bacteremia caused by MDR Gram + organisms; (**D**) Changes in the incidence of bacteremia caused by all MDR organisms; (**E**) Changes in the incidence of other infections caused by MDR Gram + organisms; (**F**) Changes in the incidence of other infections caused by MDR Gram − organisms. Abbreviations: Carba-R, carbapenem-resistant; Gram−, Gram-negative organisms; Gram+, Gram-positive organisms

The effects of the ASP on infections caused by MDR organisms in the ICU are presented in Table 3; Fig. 2. Before the intervention, the incidence of bacteremia caused by all MDR organisms (β1: 0.02 events/100 PD, *p* = 0.031) and MDR Gram-positive organisms (β1: 0.01 events/100 PD, *p* < 0.001) showed a significant upward trend. In contrast, the incidence of bacteremia due to MDR Gram-negative organisms, carbapenem-resistant Gram-negative organisms, and other infections caused by MDR organisms exhibited non-significant baseline trends.

After the intervention, the incidence of bacteremia caused by all MDR organisms (β3: −0.03 events/100 PD, *p* = 0.036) and MDR Gram-positive organisms (β3: −0.01 events/100 PD, *p* = 0.002) showed a significant downward trend. A non-significant decline was observed in the incidence of bacteremia due to MDR Gram-negative organisms and carbapenem-resistant Gram-negative organisms, as well as in the incidence of other infections caused by MDR organisms.

**Table 3:** Effect of the ASP intervention on infections due to MDR organisms

Microbiological Outcomes	Parameter evaluated	Effect of intervention	Standard Error	t	*P*	95% CI
MDR Gram- bacteremia	Pre-Intervention Trends (β1)	0.01	0.01	1.14	0.268	(− 0.01,0.03)
	Immediate Level Changes (β2)	−0.03	0.10	−0.32	0.753	(− 0.24,0.18)
	Pro-Intervention Trends (β3)	−0.02	0.01	−1.21	0.239	(− 0.05,0.01)
Carba-R Gram- bacteremia	Pre-Intervention Trends (β1)	0.01	0.01	0.92	0.370	(− 0.01,0.02)
	Immediate Level Changes (β2)	0.03	0.06	0.50	0.624	(− 0.10,0.16)
	Pro-Intervention Trends (β3)	−0.02	0.01	−1.90	0.073	(− 0.04,0.00)
MDR Gram + bacteremia	Pre-Intervention Trends (β1)	0.01	< 0.01	4.42	0.000	(0.00,0.02)
	Immediate Level Changes (β2)	−0.09	0.02	−4.15	0.000	(− 0.13,−0.04)
	Pro-Intervention Trends (β3)	−0.01	< 0.01	−3.56	0.002	(− 0.02,0.00)
ALL MDR bacteremia	Pre-Intervention Trends (β1)	0.02	0.01	2.32	0.031	(0.00,0.04)
	Immediate Level Changes (β2)	−0.11	0.09	−1.25	0.227	(− 0.29,0.07)
	Pro-Intervention Trends (β3)	−0.03	0.01	−2.25	0.036	(− 0.05,0.00)
MDR Gram + Other sites *	Pre-Intervention Trends (β1)	0.01	0.011	0.79	0.442	(− 0.01,0.022)
	Immediate Level Changes (β2)	−0.01	0.07	−0.19	0.855	(− 0.15,0.12)
	Pro-Intervention Trends (β3)	−0.02	0.01	−1.68	0.109	(− 0.04,0.00)
MDR Gram- Other sites **	Pre-Intervention Trends (β1)	0.04	0.04	1.22	0.236	(− 0.03,0.12)
	Immediate Level Changes (β2)	−0.21	0.31	−0.69	0.500	(− 0.86,0.44)
	Pro-Intervention Trends (β3)	−0.03	0.05	−0.67	0.513	(− 0.13,0.07)

*MDR* multidrug-resistant *Carba-R* carbapenem-resistant, *Gram-* Gram-negative organisms, *Gram+* Gram-positive organisms

*The source of specimens from other sites of MDR Gram+: pleural and peritoneal effusion, skin secretions, and lower respiratory tract secretions, **The source of specimens from other sites of MDR Gram-: pleural and peritoneal effusion, urine, lower respiratory tract secretions, cerebrospinal fluid, and sputum

### Mean length of stay and patient mortality rates

Table 4 shows no significant pre-intervention trends in mortality (β1: < −0.01 all-cause deaths/100 PD, *p* = 0.993), hospital stay (β1: −0.11 days, *p* = 0.567), and ICU stay (β1: 0.23 days, *p* = 0.343). Post-intervention, non-significant trends were also observed in mortality rates (β3: 0.02 all-cause deaths/100 PD, *p* = 0.216), hospital stay (β3: 0.25 days, *p* = 0.282), and ICU stay (β3: 0.06 days, *p* = 0.847).

**Table 4:** Effect of the ASP intervention on mortality, mean length of hospital stay and mean length of ICU stay in the ICU

Secondary Outcomes	Parameter evaluated	Effect of intervention	Standard Error	t	*P*	95% CI
Mortality	Pre-Intervention Trends (β1)	<−0.01	0.01	−0.01	0.993	(− 0.02,0.02)
	Immediate Level Changes (β2)	−0.03	0.09	−0.36	0.720	(− 0.23,0.16)
	Pro-Intervention Trends (β3)	0.02	0.01	1.28	0.216	(− 0.01,0.04)
Length of hospital stay	Pre-Intervention Trends (β1)	−0.11	0.18	−0.58	0.567	(− 0.49,0.28)
	Immediate Level Changes (β2)	−0.64	1.62	−0.39	0.699	(− 4.02,2.74)
	Pro-Intervention Trends (β3)	0.25	0.23	1.11	0.282	(− 0.22,0.72)
Length of ICU stay	Pre-Intervention Trends (β1)	0.23	0.24	0.97	0.343	(− 0.26,0.72)
	Immediate Level Changes (β2)	−2.28	2.08	−1.10	0.286	(− 6.63,2.06)
	Pro-Intervention Trends (β3)	0.06	0.29	0.20	0.847	(− 0.56,0.67)

## Discussion

This study evaluated the effects of an ASP in Chinese ICUs using a dual-track strategy: intensive prospective preauthorization for high-risk antimicrobials (carbapenems), and RPAF for others. This tiered approach significantly reduced overall antibiotic consumption (particularly carbapenems and linezolid). It also lowered the incidence of bloodstream infections caused by MDR organisms. Importantly, these outcomes were achieved without significantly increasing patient mortality or prolonging hospital or ICU stay, supporting the safety of restrictive measures for high-priority antimicrobials.

A key innovation of this study is the delegation of preauthorization authority to clinical pharmacists. While pharmacists commonly support ASPs worldwide, our study is the first to formalize clinical pharmacist-led preauthorization in Chinese ICUs, shifting their role from advisory to decisional. Traditionally, carbapenem prescriptions in China are approved by clinical department heads or administrators. However, physicians often lack the time for thorough reviews due to resource constraints, and hospital administrators typically lack the pharmaceutical expertise to evaluate antibiotic appropriateness. This study addressed these challenges by granting trained clinical pharmacists autonomous authorization power, after competency certification in areas such as infectious disease management and susceptibility interpretation. This represents an institutional innovation in China’s healthcare system, demonstrating that clinical pharmacist-led stewardship can maintain patient safety while easing physician workload.

Given the relatively mild pre-intervention increase in the consumption of non-carbapenem antibiotics, a less intensive intervention (RPAF), which proved effective, was applied for them. The implementation of the ASP was associated with a significant reduction in linezolid consumption, which may be attributable to several factors. First, the non-significant downward trend in non-bacteremic MDR Gram-positive infections likely decreased clinical demand. This trend may, in turn, be associated with the decrease in third- and fourth-generation cephalosporin and fluoroquinolone use [23–30], likely attributable to RPAF. Second, pharmacist-led education improved prescriber awareness that linezolid and glycopeptides are inappropriate for methicillin-sensitive *Staphylococcus aureus* infections.

However, combined analysis of glycopeptides and linezolid—both used against resistant Gram-positive pathogens—showed no significant reduction in overall use of anti-Gram-positive agents post-intervention. This suggests that prescribing shifted from linezolid to vancomycin, likely due to the recognition of linezolid’s bacteriostatic limitations in bacteremia [31], rather than an actual reduction in the overall utilization of Gram-positive agents. The non-significant increase in vancomycin use warrants pharmacovigilance given its risk of nephrotoxicity, and should be a future target for ASP optimization.

Although an increase in β-lactam/β-lactamase inhibitor combination use was observed, it was not statistically significant. This trend highlights a potential limitation of the preauthorization approach: prescribers may substitute restricted agents with alternatives of similar spectrum, creating a substitution effect. While RPAF could theoretically mitigate this issue, current evidence is insufficient to confirm its effectiveness.

Many hospitals in China continue to face challenges in establishing well-defined ASP strategies, particularly with respect to carbapenem antibiotics. This study investigated the effectiveness of ASPs in China’s unique healthcare sector, reporting novel evidence that delegating preauthorization review authority to clinical pharmacists enhances the efficiency of preauthorization processes. Furthermore, combining preauthorization with RPAF ensured a refined approach to antibiotic management and rational use of antibiotics. This approach demonstrated both feasibility and effectiveness, offering a scalable blueprint for other ICUs in China and beyond, particularly in resource-limited settings with physician shortages and high rates of antibiotic resistance. By adopting a tiered intervention strategy—reserving intensive preauthorization for high-priority antibiotics and using RPAF for others—institutions can achieve meaningful reductions in antibiotic consumption and MDR without compromising patient safety or outcomes.

Nonetheless, the ASP model implemented in this study requires a substantial allocation of administrative resources, including sustained involvement of specialized personnel, data monitoring, and analysis. Future research should investigate how to optimize these processes, integrate digital tools such as Clinical Decision Support Systems (CDSS), and develop more efficient intervention models targeting high-risk patients or specific antibiotic classes. Such approaches could reduce resource demands and enable broader, lower-cost ASP implementation.

## Conclusion

The implementation of a multidisciplinary, tiered ASP in a Chinese ICU–featuring prospective preauthorization for high-risk antibiotics such as carbapenems and retrospective audit and feedback for other agents–resulted in significant reductions in overall antibiotic consumption, particularly of carbapenems and linezolid, and a decreased incidence of bacteremia caused by multidrug-resistant organisms. Importantly, these benefits were achieved without compromising patient safety, as indicated by unchanged mortality rates and lengths of hospital and ICU stay. A key innovation of this ASP was the formal delegation of preauthorization authority to trained clinical pharmacists, enhancing review efficiency and integrating specialized pharmaceutical expertise into stewardship decisions. This model demonstrates that a structured, clinical pharmacist-supported ASP is both feasible and effective in the Chinese ICU context, providing a practical framework for promoting rational antibiotic use and curbing antimicrobial resistance in similar healthcare settings.

## Supplementary Information

Supplementary Material 1.

Supplementary Material 2.

## Data Availability

All data supporting this study are included in the article and its supplementary materials.
