# Supplementary material for: Evaluation of an antibiotic stewardship program for promoting rational antibiotic use in an ICU in China
Source: BMC Infect Dis. 2025 Oct 14;25:1301. doi: 10.1186/s12879-025-11718-4 (PMC12523123; doi:10.1186/s12879-025-11718-4)
Supplement: Supplementary file 1 — Supplementary Material 1. [file 12879_2025_11718_MOESM1_ESM.docx]

**Supplementary Table 1**

Patient characteristics in the intensive care unit during the pre- and post-intervention periods

| Characteristic | Pre-intervention period | Post-intervention period | t/Z | *p-value* |
| --- | --- | --- | --- | --- |
| Admissions | n=862 | n=946 |  |  |
| Males, n (%) | 478(55.45) | 543 (57.4) | 0.606 | 0.436 |
| Age, M (Q1, Q3) | 64(53~74) | 65 (54~75) | −1.238 | 0.216 |
| weight, M (Q1, Q3) | 65(55~70) | 65 (60~70) | −0.102 | 0.918 |
| Nutrition Assessment Score, M (Q1, Q3) | 3(3~4) | 3 (3~4) | −0.037 | 0.970 |
| Acute Physiology and Chronic Health Evaluation II score, M (Q1, Q3) | 17(12.75~22) | 17 (12~22) | −0.776 | 0.438 |
| Patients with main diagnosis of infection, n (%) | 278(32.25) | 324 (34.25) | 0.811 | 0.368 |
| Patients who underwent any surgical procedure*, n (%) | 106(12.30) | 137 (14.48) | 1.851 | 0.174 |
| Patients with neoplastic disease, n (%) | 34(3.94) | 25 (2.64) | 2.421 | 0.120 |
| Patients with diabetes, n (%) | 153(17.75) | 165 (17.44) | 0.029 | 0.864 |
| Patients with chronic lung disease**, n (%) | 95(11.15) | 90 (9.51) | 1.301 | 0.254 |
| Patients using ventilators, n (%) | 374(43.39) | 401 (42.39) | 0.184 | 0.668 |

*Abdominal, thoracic, or urological surgery; **Chronic obstructive pulmonary disease, bronchiectasis, emphysema
