# Supplementary material for: Evaluation of an antibiotic stewardship program for promoting rational antibiotic use in an ICU in China
Source: BMC Infect Dis. 2025 Oct 14;25:1301. doi: 10.1186/s12879-025-11718-4 (PMC12523123; doi:10.1186/s12879-025-11718-4)
Supplement: Supplementary file 2 — Supplementary Material 2. [file 12879_2025_11718_MOESM2_ESM.docx]

**Supplementary Table 2**

Facility-specific criteria for carbapenems

| Imipenem/Cilastatin | Meropenem |
| --- | --- |
| Bloodstream infections caused by ESBL-producing bacteria | Bloodstream infections caused by ESBL-producing bacteria |
| Non-bloodstream but severe infections caused by ESBL-producing bacteria | Non-bloodstream but severe infections caused by ESBL-producing bacteria |
| Infections that are non-bloodstream but severe, caused by bacteria suspected of producing ESBLs with prior treatment failure, despite adequate dosage and duration of therapy by β-lactam/β-lactamase inhibitor combinations | Infections that are non-bloodstream but severe, caused by bacteria suspected of producing ESBLs with prior treatment failure, despite adequate dosage and duration of therapy by β-lactam/β-lactamase inhibitor combinations |
| Infections that are non-bloodstream but severe, caused by bacteria suspected of producing ESBLs with β-lactam/β-lactamase inhibitor combinations allergy | Infections that are non-bloodstream but severe, caused by bacteria suspected of producing ESBLs but allergy to β-lactam/β-lactamase inhibitor combinations |
| septic shock | septic shock |
| Agranulocytosis with fever and pathogen unknown with high risk for serious complications[23] and risk factors for drug resistance[24] | Agranulocytosis with fever and pathogen unknown with high risk for serious complications and risk factors for drug resistance |
|  | Severe infections of the central nervous system caused by bacteria suspected of producing ESBLs |
|  | Severe infections of the central nervous system with β-lactam allergy |

ESBLs: extended-spectrum beta-lactamases
